# Clinical characteristics and outcome of patients aged over 80 years with covid-19

**DOI:** 10.1097/MD.0000000000024750

**Published:** 2021-02-26

**Authors:** Aina Capdevila-Reniu, Martina Pellice, Sergio Prieto-González, Helena Ventosa, Andrea Ladino, Jose Naval, Olga Rodriguez-Nuñez, Jose César Milisenda, Pedro Juan Moreno-Lozano, Alex Soriano, Xavier Bosch, Alfonso López-Soto

**Affiliations:** a Department of Internal Medicine; b Department of Infectious Diseases, Hospital Clínic of Barcelona, Institut d’Investigacions Biomèdiques August Pi i Sunyer (IDIBAPS), University of Barcelona, Spain.

**Keywords:** covid-19, elderly, illness severity, mortality

## Abstract

To investigate the clinical characteristics and outcome of octogenarians with covid-19.

This is a observational, retrospective, descriptive study.

Consecutive patients aged >80 years who were admitted for covid-19 pneumonia during a 6 weeks period (March 20–April 30, 2020).

Illness severity on admission was classified according to World Health Organization (WHO) criteria: mild, moderate, severe, and critical. Data collected included demographics, presenting symptoms, radiological and laboratory findings, comorbidities, functional status, treatment, and clinical outcome.

There were 159 patients (52.2% women) with a median age of 85.99 (IQR: 80–98). The median Barthel index was 90 (40–100) and Charlson index was 5 (5–6). Most common presenting symptoms were fever, dyspnea, and cough. Patients had mild (8.2%), moderate (52.2%), or severe (39.6%) illness according to WHO criteria. A bilateral pulmonary involvement was seen in 86% of patients. Laboratory analysis revealed increased serum concentrations of inflammatory parameters (C-reactive protein, ferritin, lactate dehydrogenase, and D-dimer) with an abnormal lymphocyte count [0.88 × 10^9^/L (0.5)]. Treatments included corticosteroids in 37%, and biological therapies in 17.6%. Fifty three (33.3%) patients died during hospitalization, with a median time from admission to death of 3 (IQR 1–6) days. Mortality was higher in men (55%). Deceased patients had a significantly higher frequency of dyspnea, increased inflammatory parameters, and illness severity compared to survivors.

One-third of octogenarians with covid-19 died during hospitalization and most had bilateral lung involvement. A further knowledge of the characteristics and outcome of this population may assist clinicians in the decision-making process in these patients.

## 1 Introduction

After causing nearly 2 million deaths and unprecedented social and economic disruptions, the coronavirus disease 2019 (covid-19) pandemic might come to an end in 2021 if recently authorized vaccines for active immunization on a global scale prove, as expected, effective. Although specific therapeutic options for the causative agent of covid-19, that is, severe acute respiratory syndrome coronavirus-2 (SARS-CoV-2), a novel coronavirus first identified in December 2019 in China,^[1]^ are not available or are undergoing clinical trials, recent research has yielded promising results. Particularly, studies investigating quinoline-based inhibitors against SARS-CoV-2 have shown that a novel phytocompound (quinoline,1,2,3,4-tetrahydro-1-[(2-phenylcyclopropyl)sulfonyl]-trans-(8CI)) is a potent inhibitor of the main proteases of the virus and, although it needs to be synthesized first, could become a new drug for the treatment of covid-19.^[2]^

In Spain, the first case of covid-19 was reported in late January 2020 and there was an exponential increase in the reported rate of infections in the ensuing weeks. It was not until May that, after a national lockdown was imposed in mid-March, the number of new infections and deaths decreased progressively.^[3]^

The old population has been the most affected group during the global pandemic, with high mortality rates. Current estimates indicate that more than 7 million Spaniards are aged over 65 years and approximately 25% of these are over 80.^[4]^

By April 30, 2020, the number of confirmed cases of covid-19 in Spain was 203,715, with 48,916 cases being reported in the region of Catalonia. Among 81,627 reported admissions in Spanish hospitals, 21,306 (26.1%) corresponded to patients aged over 80 years. Of note, a mortality rate of 86% has been reported in those older than 70 years.^[5]^ From February 18 (upon confirmation of the first case) to April 18, 2337 patients were hospitalized at the Hospital Clínic of Barcelona, which would correspond to 2.86% of all admissions in Spanish hospitals.^[5]^

The aging process is characterized by important physiological changes which bring about impaired responses to external agents. It is well-known that older patients may present with clinical manifestations that are often attenuated or even absent (e.g., fever). Because of greater comorbidity and poorer immune responses, elderly patients are the most vulnerable population group.^[6]^

Detecting, monitoring, and treating high risk groups of covid-19 patients such as the older ones is essential to decrease the mortality rates. However, to date, there are no studies on covid-19 focusing on older patients in their 80 seconds or 90 seconds. This observational, retrospective study was aimed at investigating the clinical characteristics, course and treatment of patients aged over 80 years who were admitted in the Hospital Clínic with covid-19. We also sought to analyze the differences between deceased and recovered patients and how different factors correlated with changes in treatment over time.

## 2 Methods

### 2.1 Study participants and data collection

We included all consecutive patients aged over 80 years who were admitted for covid-19 pneumonia at the Hospital Clinic of Barcelona during a 6-week period (from March 20 to April 30, 2020). Hospital Clinic is a university, tertiary center with a reference population of 550,000 and an occupation of 713 beds for acute patients. As patient volume increased during the pandemic, medicine bed capacity was expanded, with approximately 85% of acute beds intended for covid-19 patients at the pandemic outbreak in Spain. The Institutional Ethics Committee of the Hospital Clínic of Barcelona approved the study and due to the nature of retrospective chart review, waived the need for inform consent from individual patients.

Most covid-19 patients were diagnosed based on a positive result on the reverse transcriptase polymerase chain reaction (PCR) assay for SARS-CoV-2. Some patients in whom a PCR assay was not performed were considered to have covid-19 on the basis of clinical, laboratory and radiological criteria upon the existing epidemiological context once other infections were ruled out.

Patients’ disease severity on admission was classified according to World Health Organization (WHO) criteria: mild (without pneumonia), moderate (presence of pneumonia, no signs of severe pneumonia and no need for supplemental oxygen), severe (fever or suspected respiratory infection, plus one of: dyspnea, respiratory frequency ≥30/minute, blood oxygen saturation ≤93%, PaO_2_/FiO_2_ ratio 50% of the lung field within 24–48 hours), and critical [respiratory failure that requires mechanical ventilation and shock or organ failure that requires admission to the intensive care unit (ICU)].^[7]^

Owing to the reduction of resources of the ICU during the pandemic outbreak, the criteria of patients transfer to it were redefined to offer this resource to patients with greater survival capacity. In this context, following the recommendations of the Ethics Committee of the Hospital Clínic, age, comorbidities, and geriatric evaluation were considered key parameters to limit the access to intensive care but not for semicritical interventions.

Data were manually abstracted from the Hospital Clinic electronic health record. Data collected included demographics, presenting signs and symptoms, and radiological and laboratory findings. Comorbidities were assessed individually and through determination of the Charlson comorbidity index. Only previously documented cognitive impairment was registered. Patients underwent a geriatric evaluation through determination of the Barthel index. We analyzed treatments received and clinical evolution of each case including mortality during hospitalization, transfer to the ICU, complications such as acute respiratory distress syndrome or acute kidney injury, use of mechanical ventilation, length of stay, and disposition (transfer, discharge, or death). All data were collected by a medical team trained in the healthcare of covid-19 patients. We excluded from the analysis data that were not found in the electronic health record and no imputation was performed.

### 2.2 Statistical analysis

Absolute numbers and percentages were used for categorical variables, mean [standard deviation (SD)] for normally distributed continuous variables, and median [interquartile range (IQR) 25–75] for non-parametric data. Categorical variables were compared with the χ^2^ test and normally distributed continuous variables with the *t* test; otherwise, the Mann–Whitney test was used. We also performed subgroup analyses to compare deceased and recovered patients. Multivariate logistic regression analysis was performed to detect potential predictors of mortality. Variables with a *P* value <.20 in the univariate analysis were included in the multivariate analysis. The presence of interaction and the role of confounding factors were evaluated; statistical significance was accepted at a 5% level. We used SPSS-v.25 for all analyses.

### 2.3 Patient and public involvement statement

The quick progression and emergency of the covid-19 pandemic outbreak meant a need to quickly disseminate information. Therefore, patients were not directly involved in the design, interpretation, or development of this study. In addition, this was a retrospective case series study and nonresearch or nonmedical personnel had no access to chart reviews, thus limiting public and patient involvement.

## 3 Results

During the 6 weeks period of the study, 159 patients aged ≥80 years who were admitted to the hospital with covid-19 were discharged from hospital or dead. The PCR assay for SARS-CoV-2 confirmed the diagnosis in 109 (69%) of these patients.

Table 1 shows the baseline characteristics of all patients included. Mean age was 86 (4.1) years and 52.2% were women. The median Barthel index was 90 (40–100) and the median Charlson comorbidity index was 5 (5–6). Main comorbidities included hypertension (75.6%), dementia (30%), type 2 diabetes mellitus (29%), chronic kidney disease (26%), and coronary heart disease (16%).

**Table 1:** Baseline characteristics of 159 patients aged ≥ 80 years with covid-19.

Characteristics	n (%)	
Gender, n (%)	Men: 76 (48) Women: 83 (52)	
Age, years, mean (SD)	86 (4.1)	
Barthel index, median (IQR)	90 (40–100)	
Dementia, n (%)	50 (31)	
Charlson comorbidity index, median (IQR)	5 (5–6)	
Diabetes mellitus, n (%)	46 (29)	
Chronic obstructive pulmonary disease, n (%)	17 (10.7)	
Cerebral vascular disease, n (%)	18 (11.3)	
Coronary heart disease, n (%)	25 (16)	
Cancer, n (%)	21 (13)	
Chronic kidney disease, n (%)	41 (26)	
Hypertension, n (%)	120 (75.5)	
Symptoms on admission, n (%)	Fever: 118 (74)	
	Cough: 68 (43)	
	Dyspnea: 80 (50.3)	
	Diarrhea: 19 (12)	
	Anosmia: 3 (2)	
	Odynophagia: 4 (3)	
	Ageusia: 10 (6.3%)	
	Headache: 3 (2)	
	Falls: 13 (8.2)	
Classification of severity (World Health Organization), n (%)	Mild: 13 (8.2)	
	Moderate: 83 (52.2)	
	Severe: 63 (39.6)	
Place of origin, n (%)	Home: 104 (65.4)	
	Nursing home: 55 (35)	
Radiological pattern, n (%)	No pneumonia: 12 (7.5)	
	Pneumonia 147 (92.5)	Unilateral lung involvement: 20 (14)
		Bilateral lung involvement: 127 (86)
Laboratory tests, mean (SD)	CRP: 10.7mg/dl (9.2)	
	LDH: 337 U/L (152)	
	Ferritin: 694 ng/ml (879)	
	Lymphocyte count: 0.88 x10^9^/L (0.5)	
	D-dimer: 2777 ng/ml (3982)	
Treatment during hospitalization, n (%)	Triple treatment: 83 (78) ^∗^	
	Corticosteroids (mg/kg): 59 (37)	
	Biological therapies: 28 (17.6)	
Deaths, n (%)	53 (33.3)	
Days until hospital admission, median (IQR)	5 (2–8)	

CRP = C-reactive protein, IQR = interquartile range, LDH = lactate dehydrogenase, SD = standard deviation, WHO = World Health Organization.

∗ Triple treatment: Lopinavir/Ritonavir, Hidroxicloroquine, and Azitromicine.

Most common symptoms on presentation were fever (74.2%), dyspnea (50.3%), and cough (42.8%), while less frequent were diarrhea (12%), ageusia (5.3%), and anosmia (2%). An 8% of patients had suffered a fall from their own height the day before admission. The median time from onset of symptoms to hospital admission was 5 (2–8) days.

According to WHO criteria, 13 (8.2%) patients were initially classified as having a mild illness severity, 83 (52.2%) patients moderate, and 63 (39.6%) patients as severe.^[5]^ When evaluating chest radiographs of all patients on admission, 92.5% had pulmonary involvement and of those 86% had bilateral involvement.

Laboratory analysis on presentation showed increased concentrations of serum inflammatory markers. In particular, mean levels of C-reactive protein (CRP) and ferritin were 10.7 mg/dl (9.2) and 694 ng/ml (879), respectively. Moreover, the mean serum level of lactate dehidrogenase was 337 U/L (152) and D-dimer 2777 ng/ml (3982), and the mean lymphocyte count was 0.88 × 10^9^/L (0.5).

The most commonly administered treatment in 74.8% of patients was a pharmacological combination of lopinavir/ritonavir (200/50 mg every 12 hours for 7–14 days), hydroxychloroquine (400 mg every 12 hours the first day and subsequently 200 mg every 12 hours, for a total of 5 days) and azithromycin (500 mg the first 24 hours and then 250 mg every 24 hours for a total of 5 days). This pharmacological combination was incomplete in 38% of cases due to adverse effects (oral intolerance mostly diarrhea, ionic abnormalities, and QT lengthening on the electrocardiogram) that led to withdrawal of some of the drugs. No patient presented severe adverse effects resulting from this combined treatment. Furthermore, 37% of patients received corticosteroids at different doses and 17.6% received biological therapies. Biologics used included tocilizumab in 13 patients, baricitinib in 12, anakinra in 6, and siltuximab in 1 patient. Side effects of biologic agents were limited to transient elevations of liver enzymes in 4 patients. There were no infections associated with the use of these therapies.

Fifty three (33.3%) patients died during their hospitalization, with a median time from admission to death of 3 days (1–6) days. Mortality was higher in men (55%) compared to women (45%). The main differences between deceased and recovered patients with covid-19 are shown in Table 2. Age, comorbidities, and previous functionality were similar between the 2 groups. Deceased patients had a significantly higher frequency of dyspnea, increased inflammatory parameters, and illness severity compared to survivors. On multivariate logistic regression, (Table 3), we found that mortality was independently associated with the severity of disease on admission (OR = 4.36; 95% CI [1.21–8.93]; *P* = .02), history of prior dyspnea (OR = 3.28; 95% CI [1.18–11.22]; *P* = .025) and CRP on admission (OR = 1.01; 95% CI [1.23 – 1.82]; *P* = .01).

**Table 2:** Comparison of surviving patients during hospital admission and died patients due to SARS-CoV-2.

	Recovered (n = 106)		Dead (n = 53)		*P* value
Gender, n (%)	Men: 47 (44)		Men: 29 (55)		.22
	Women: 59 (56)		Women: 24 (45)		.21
Age, years, mean (SD)	86 (4.2)		86 (4.0)		.96
Barthel index, median (IQR)	95 (60–100)		65 (40–100)		.06
Dementia, n (%)	32 (30)		8 (15)		.63
Charlson comorbidity index, median (IQR)	5 (5–6)		6 (5–6)		.50
Diabetes mellitus, n (%)	29 (27)		17 (25)		.54
Chronic obstructive pulmonary disease, n (%)	13 (12)		4 (8)		.36
Cerebral vascular disease, n (%)	11 (10)		7 (13)		.59
Coronary heart disease, n (%)	15 (14)		10 (19)		.44
Cancer, n (%)	11 (10)		10 (19)		.14
Chronic kidney disease, n (%)	25 (24)		16 (30)		.37
Hypertension, n (%)	81 (76)		39 (74)		.70
Symptoms on admission, n (%)	Fever: 78 (74)		Fever: 40 (75)		.80
	Cough: 50 (47)		Cough: 18 (34)		.11
	Dyspnea: 40 (38)		Dyspnea: 40 (75)		**.001**
	Diarrhea: 16 (15)		Diarrhea: 3 (6)		.08
	Anosmia: 3 (3)		Anosmia: 0 (0)		.55
	Odynophagia: 3 (3)		Odynophagia: 1 (2)		1
	Ageusia: 10 (9)		Ageusia: 0 (0)		**.03**
	Headache: 3 (5.5)		Headache: 0 (0)		.55
	Falls: 11 (10)		Falls: 2 (4)		.22
Classification of severity (WHO), n (%)	Mild: 11 (10)		Mild: 2 (3)		.22
	Moderate: 71 (67)		Moderate: 12 (23)		**.001**
	Severe: 24 (23)		Severe: 39 (74)		**.001**
Place of origin, n (%)	Home: 73 (69)		Home: 31 (58)		.19
	Nursing home: 33 (31)		Nursing home: 22 (42)		.19
Radiological pattern, n (%)	No pneumonia: 10 (9)		No pneumonia: 2 (4)		.34
	Pneumonia 90 (91)	Unilateral lung involvement: 14 (13)	Pneumonia 51 (96)	Unilateral lung involvement: 6 (11)	.73
		Bilateral lung involvement: 82 (77)		Bilateral lung involvement: 45 (85)	.26
Laboratory tests, mean (SD)	CRP: 7.8 mg/dl (6.3)		CRP: 16.4 mg/dl (11.4)		**.001**
	LDH: 306 U/L (108)		LDH: 403 U/L (204)		**.001**
	Ferritin: 551 ng/ml (490)		Ferritin: 971 ng/ml (1313)		**.013**
	Lymphocyte count: 0.9 ×10^9^ L (0.5)		Lymphocyte count: 0.8 ×10^9^ L (0.5)		.10
	D-dimer: 2190 ng/ml (2780)		D-dimer: 3986 ng/ml (5555)		**.03**
Treatment during hospitalization, n (%)	Triple treatment: 83 (78) ^∗^		Triple treatment: 36 (68) ^∗^		.15
	Corticosteroids (mg/kg): 34 (32)		Corticosteroids (mg/kg): 25 (47)		.06
	Biological therapies: 21 (20)		Biological therapies: 7 (13)		.30

CRP = C-reactive protein, IQR = interquartile range, LDH = lactate dehydrogenase, SD = standard deviation, WHO = World Health Organization.

∗ Triple treatment: Lopinavir/Ritonavir, Hidroxicloroquine, and Azitromicine.

**Table 3:** Multivariate logistic analysis between surviving patients during hospital admission and died patients due to SARS-CoV-2.

Risk factor	Odds ratio	95% CI	P
CRP	1.01	1.23 – 1.82	.01
Dyspnea	3.28	1.18 – 11.22	.025
Severe disease on admission	4.36	1.21 – 8.93	.02

CI = confidence interval, CRP = C-reactive protein.

**Figure 1:**
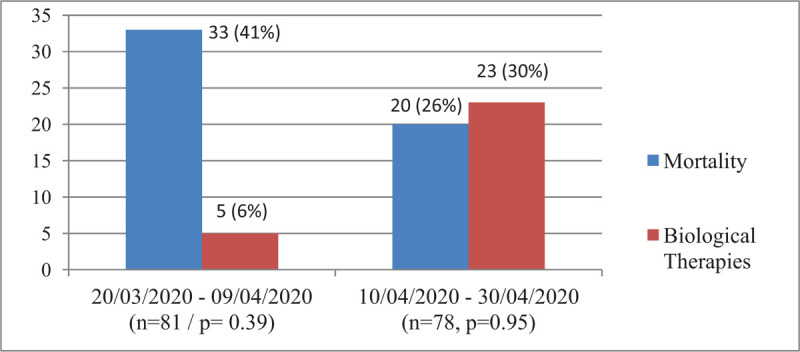
Comparison of mortality rates between the 2 three-week periods of the study.

**Figure 2:**
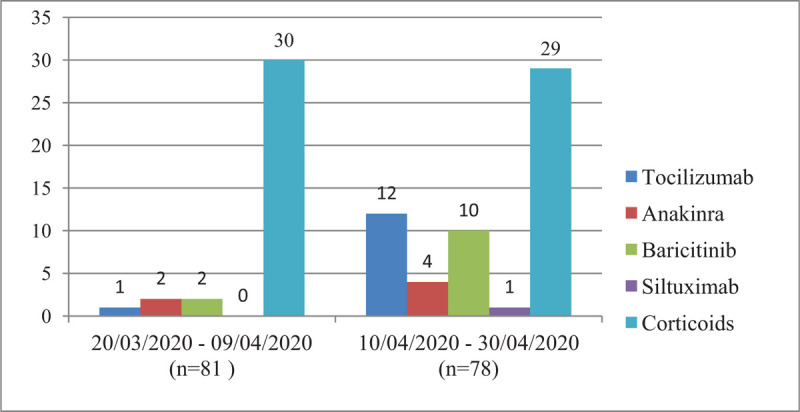
Distribution of the different immunosuppressants used between the 2 three-week periods of the study.

We further compared the mortality rates in relation to the frequency of use of biological therapies in 2 three-weeks periods: from March 20 to April 20, when 5 (4%) patients had received biological therapies, to the second half of the full study period (i.e., 10–30 April) when the total number of patients who had received biologics was 32 (20%). As shown in Figure 1, as more patients were treated with biological therapies during the last 3 weeks, a nonsignificant decrease in mortality was observed during this period. Figure 2 displays the distribution of the different biological agents, along with corticosteroids, administered during these 2 periods. In addition, no significant mortality differences were found in relation to the use of corticosteroids.

Finally, at the time of hospital discharge, 54% of patients were referred to their homes, 33% to nursing homes, and 13% were transferred to social and healthcare centers to complete their recovery process.

## 4 Discussion

Among 159 patients older than 80 years who were hospitalized during a 6-week period, one-third of them died during hospitalization and 80% had bilateral lung involvement. As far as we are aware, this represents the first study of consecutively hospitalized older patients with covid-19. The mean baseline comorbidities and functional status of our cohort did not differ significantly from those expected in elder patients aged over 80 years. Furthermore, differences did not surface when comparing deceased and recovered patients, even though there was a nonsignificant trend for a higher Barthel index in the former. Rather, the extension of lung involvement, the severity of disease based on WHO criteria, the presence of dyspnea on admission, and the pattern of serum inflammation parameters were the marked differences between the 2 groups. A better understanding of baseline characteristics and clinical outcomes of our population of aged patients may help clinicians and facilitate the medical decision-making in these patients.

### 4.1 Comparison with other studies

From February 18 to April 30, 2020 the average mortality rate among all covid-19 patients admitted to Hospital Clínic was 11% (unpublished data). Among octogenarians, this figure rose to 33.3%. There is a substantial variation in the reported percentage of hospital mortality in patients older than 60 years with covid-19, ranging from 19% to 32.7%.^[8,9]^ Mortality rates of covid-19 in the general population vary according to the region of origin, with higher figures being reported in the eastern Mediterranean region (6.67%), Asia (3.45%), and Europe (2.06%).^[10]^ Data from the Spanish Health Alerts and Emergencies Coordination Center at the end date of our study showed that the mortality in patients aged ≥80 years was 21% (n = 9727), with a higher lethality in women.^[3]^ However, a WHO report with a date of February 28, 2020, with data obtained from China, showed a crude fatality ratio of 21.9% in this age group.^[11]^ Several factors may account for this substantial data mismatch, such as the criteria for hospital admission. In line with our results, a study conducted in the New York area showed a mortality of 53.7%, in covid-19 patients older than 80 years, with higher mortality rates for men (61.3%) than for women (47.5%).^[8]^ In the same direction, in unpublished data from Hospital La Paz – Madrid, the mortality rate was 55% in patients older than 80 years and was higher in men.^[12]^ This high mortality agrees with different scientific reports showing that old age and comorbidities are the main risk factors for fatal events.^[13]^ In addition, as expected, patients initially classified as severe by WHO criteria had a higher mortality rate.

In our series, most patients had a moderate to severe clinical disease on hospital admission (according to WHO criteria), with most developing a rapid clinical progression of the disease that led to early in-hospital mortality. Both groups (recovered patients and death) had high serum levels of inflammatory parameters and a greater extension of pneumonia on admission. Zhou et al reported as potential risk factors for adverse events: an advanced age, elevated SOFA score (Sequential Organ Failure Assessment) and plasma D-dimer levels greater than 1 μg/ml.^[14]^ In Wang et al study, the presence of dyspnea on admission, a previous history of cardiovascular disease or COPD (Chronic Obstructive Pulmonary Disease), and the presence of respiratory distress were predictors of mortality, while a high number of lymphocytes was a protective factor.^[9]^ In the same publication, they described that 70% of patients older than 60 years with covid-19 had criteria of serious or critical illness (according to WHO classification) on hospital admission and the in-hospital mortality was 19%. Moreover, for patients developing ARDS (Acute Respiratory Distress Syndrome), the 28-day mortality was nearly 50%.

In our cohort, as in others, the most frequent symptoms on admission were fever, dyspnea, and cough. Although anosmia, ageusia, and odynophagia were uncommon, the high prevalence of cognitive impairment (30%) could lead to an underestimation of the prevalence of these symptoms.^[13,15,16]^

Deceased patients in our study had a rapid clinical deterioration with a median time from admission to death of only 3 days, similar to other series.^[8,9]^

### 4.2 Limitations of this study

This study has several limitations. First, data were collected from a single academic hospital and cannot be generalized to all other regions. Second, the retrospective nature of the study brings about potential errors in clinician documentation and data collection. Third, in the first months of the pandemic in Spain, many elderly patients with symptoms of SARS-CoV-2 infection were residing in nursing-homes and were treated there by general practitioners. It has been reported that 25,055 patients older than 80 years with covid-19 were never transferred to hospitals. Therefore, this may have caused a large selection bias in our study and the real mortality rate in this age group was probably higher.^[3]^ Another weakness of the study is the lack of PCR assay confirmation in 25% of covid-19 patients included. Although the small number of daily tests was the major cause accounting for this bias, the favorable epidemiological context in patients with symptoms and complementary tests compatible with covid-19 meant that these cases were reasonably attributed to positive cases of infection.^[5]^

## 5 Conclusion

Most patients older than 80 years with covid-19 had bilateral lung involvement and one-third died during hospitalization. Compared to survivors, deceased octogenarians had significantly higher levels of serum inflammatory markers and a significantly greater occurrence of dyspnea and illness severity.

## Author contributions

**Conceptualization:** Aina Capdevila-Reniu.

**Data curation:** Aina Capdevila-Reniu, Martina Pellice, Helena Ventosa, Andrea Ladino, Jose Naval.

**Formal analysis:** Aina Capdevila-Reniu, Martina Pellice, Helena Ventosa, Andrea Ladino, Jose Naval.

**Investigation:** Aina Capdevila-Reniu, Sergio Prieto-Gonzalez, Olga Rodriguez-Nuñez, JC Milisenda, Pedro Juan Moreno-Lozano, Alfonso López-Soto.

**Methodology:** Aina Capdevila-Reniu, Sergio Prieto-Gonzalez, Olga Rodriguez-Nuñez, JC Milisenda, Pedro Juan Moreno-Lozano, Alfonso López-Soto.

**Supervision:** Aina Capdevila-Reniu, Xav Bosch, Alfonso López-Soto.

**Validation:** Aina Capdevila-Reniu, Xav Bosch, Alfonso López-Soto.

**Visualization:** Sergio Prieto-Gonzalez.

**Writing – original draft:** Aina Capdevila-Reniu, Xavier Bosch.

**Writing – review & editing:** Aina Capdevila-Reniu, Martina Pellice, Sergio Prieto-Gonzalez, JC Milisenda, Pedro Juan Moreno-Lozano, Alex Soriano, Xavier Bosch, Alfonso López-Soto.
